# Supplementary material for: N-3 Long-Chain Polyunsaturated Fatty Acids, Eicosapentaenoic and Docosahexaenoic Acid, and the Role of Supplementation during Cancer Treatment: A Scoping Review of Current Clinical Evidence
Source: Cancers (Basel). 2021 Mar 10;13(6):1206. doi: 10.3390/cancers13061206 (PMC8000768; doi:10.3390/cancers13061206)
Supplement: Supplementary file 1 [file cancers-13-01206-s001.pdf]

cancers

MDPI

Supplementary Material: N-3 Long-Chain Polyunsaturated Fatty Acids, Eicosapentaenoic and Docosahexaenoic Acid, and the Role of Supplementation during Cancer Treatment: A Scoping Review of Current Clinical Evidence

Marnie Newell [1], Vera Mazurak [1], Lynne M. Postovit [2,3] and Catherine J. Field [1,*]

Table S1. Characteristics of the C reactive protein assessments included in clinical trials.

Cancer localization	Baseline values (mg/ml)	End of Treatment	Direction of change in N-3 group	Reference
		Pre or Post treatment		
Breast (Pre-chemotherapy)	CNT: 1 (0-2)<br>N-3: 1 (1-5)	CNT: Δ 17.2% (-0.16 to 91.99)<br>N-3: Δ -5.9% (-35.4 to 74.12)	Stable CRP compared to baseline; NS ↓ compared to CNT (*P*<0.06)	[12]
Breast (Post-treatment, on AI)	N-3 (high CRP):13.3 (±3.6)<br>N-3 (med CRP):7.6 (±1.4)<br>N-3 (low CRP): 4.8 (±0.5)	N-3 (high CRP): Δ -12 (±16.4)<br>N-3 (med CRP): Δ -2.2 (±3.0)<br>N-3 (low CRP): Δ 0.2 (±2.9)	N-3 group with highest baseline CRP had largest ↓ (*P*<0.02), med baseline CRP had second ↓ (*P*<0.04), lowest baseline CRP: NC.	[13]
		Stages III, IV (concurrent with chemotherapy) or Palliative		
Colorectal	149 (12 to 447)	CNT: Δ -5.0 (-182 to 109)<br>N-3: Δ 3.0 (-152 to 109)	NC	[40]

Colorectal	18.2 (±13.9)	N-3: 19.4 (±17.7)	No placebo; CRP levels at end of Wk 3 were ↑ and returned to baseline levels at Wk 9	[41]
Colorectal	CNT: 4.85 (1.01-6.33)<br>N-3: 3.39 (1.49-18.24)	CNT: Δ 24 (±30.2)<br>N-3: Δ -8.8 (±13.5)	↓ Compared to baseline (*P*<0.05) and CNT (*P*<0.04)	[32]
Colorectal	CNT: 6.4 (1.6-23.7)<br>N-3: 5.1 (4.1-12.3)	CNT: Δ 1 (-1.8 to 10.3)<br>N-3: Δ -1.4 (-4 to -0.7)	↓ Compared to baseline and CNT (*P*<0.06)	[33]
Head & Neck	CNT: 25 (±38)<br>N-3: 21 (±35)	CNT: Δ -13 (±36)<br>N-3: Δ -7 (±34)	NS	[18]
Leukemia	CNT: 16.9 (6.8-75.9)<br>N-3: 35.3 (10.2 -54.4)	CNT: Δ -13.7 (-47.4 to -1.1)<br>N-3: Δ -4.7 (-23.8 to -1.0)	↓ Compared to baseline (*P*<0.04)	[48]
Lung	580 (67-1134)	CNT: Δ -22 (-871 to 103)<br>N-3: Δ -45 (-1066 to 302)	NS trend towards ↓ CRP	[40]
Lung	N-3: 33 (±17.8)<br>N-3+chemo: 38 (±22.3)	N-3: Δ -6.7 (4.5)<br>N-3+chemo: -21.3 (7)	Both groups ↓ CRP to baseline (*P*<0.02); N-3+chemo ↓ CRP compared to N-3 alone (*P*<0.005)	[52]
Lung (NSCLC)	CNT: 50.4 (±66.8)<br>N-3: 39.1 (±42.9)	Not defined, but both groups decreased until study end	No difference, between groups, but malnourished had ↑ CRP compared to nourished	[53]
Lung (NSCLC)	CNT: 37 (±40)<br>N-3: 32 (±40)	CNT: Δ 1.9 (±0.2)<br>N-3: Δ -13	↓ Compared to baseline (*P*<0.02) and CNT (*P*<0.07)	[54]
Lung (NSCLC)	CNT: 11.5<br>N-3: 12.89	CNT: 27.09<br>N-3: 10.09	Stable CRP compared to control	[50]

Lung (NSCLC)	CNT: 12.7 (±3.5)<br>N-3: 13.5 (±3.9)	CNT: Δ 9.4 (6.8 to 11.3)<br>N-3: Δ -2.2 (-5.7 to -0.8)	↓ Compared to baseline and CNT (*P*<0.01)	[51]
Pancreatic	CNT: 11 (5-30)<br>N-3: <5 (<5-21)	CNT: Δ ~30% ↑<br>N-3: NC	Stable CRP compared to control	[59]
		Surgical interventions		
Esophageal (O-III)	CNT: 6 (5)*<br>N-3: 6 (5)	CNT: Surgery: 137 (45) to 54 (50) post<br>N-3: Surgery: 148 (36) to 30 (23) post	↑ after surgery & ↓ by day 21 but not different from CNT	[46]
Gastrointestinal	CNT: 0.3 (±0.1)<br>N-3: 0.4 (±0.1)	CNT: Surgery: 4.6 (±1.8) to 3.9 (±2.3) post<br>N-3: Surgery: 4.8 (±2.3) to 2.0 (±1.1) post	↑ after surgery & ↓ compared CNT by day 8 (*P*<0.05)	[24]

*Reported mg/l in this study
